# Transformation of Cardiology and Cardiothoracic Services at Benjamin Mkapa Hospital, Tanzania: Findings and Experiences from 1,313 Cardiovascular Procedures in Five Years

**DOI:** 10.5334/gh.1488

**Published:** 2025-10-17

**Authors:** Anwar Ahmed Salim, Alfred Luvakule, Hindu Ibrahim, Sanun Ally Kessy, Shemsa Khatib, Monica Kessy, Baraka Alphonce, Happiness Kusima, Kelvin Masava, Shija Kessy, Ahmed Toure, Alphonce Chandika, John Meda, Abel Makubi

**Affiliations:** 1 Department of Cardiology and Cardiothoracic Services, Benjamin Mkapa Hospital, Tanzania; 2 Directorate of Training and Research, Benjamin Mkapa Hospital, Tanzania; 3 Department of Internal Medicine, School of Medicine and Dentistry, University of Dodoma, Tanzania; 4 Department of Nephrology and Urology, Benjamin Mkapa Hospital, Tanzania; 5 Directorate of Curative Services, Ministry of Health, Tanzania

**Keywords:** CVD, cardiovascular care, health system, LMIC, Benjamin Mkapa hospital, Tanzania

## Abstract

**Background:**

Cardiovascular care remains limited in sub-Saharan Africa. Since its establishment in 2015, the Benjamin Mkapa Hospital (BMH) in Dodoma, Tanzania, has gradually strengthened its cardiology and cardiothoracic services through the integration of high-tech diagnostics, interventional procedures, and surgical capabilities, aiming to meet the growing demand for advanced medical care in Tanzania.

**Objective:**

To describe the evolution of cardiology and cardiothoracic services at the BMH, assess performance and challenges, and report on procedures conducted between 2019 to 2024.

**Methodology:**

A retrospective descriptive analysis was conducted on patient document review and the hospital electronic database between February 2019 and August 2024. The study included both pediatric and adult patients who received care in the cardiology and cardiothoracic department.

**Results:**

The transformation of cardiovascular services at the BMH from 2018 resulted in providing advanced cardiovascular care to patients in central Tanzania. A total of 1,313 procedures were performed, including 1,215 adult cardiac catheterization procedures (1,081 diagnostic coronary angiographies, 115 percutaneous coronary interventions, and 19 pacemaker implantations), 55 pediatric cardiac catheterization procedures (16 right heart catheterizations, 10 atrial septal defect device closures, 18 patent ductus arteriosus device closures, and 11 pulmonary valve valvuloplasty), and 43 open-heart surgeries, consisting of 36 congenital heart disease repairs, two valve replacements, and five coronary artery bypass grafts. Among 115 patients who underwent percutaneous coronary intervention, four died, yielding a success rate of 96.5%. Of the 43 patients who underwent open-heart surgery, three deaths were recorded, resulting in a success rate of 93.0%. These deaths were mainly due to advanced disease and surgical complications.

**Conclusion:**

The experience underscores the importance of strategic investment, leadership, and partnerships in advancing health system resilience and equity in low-resource settings. A total of 1,313 patients benefited from minimally invasive procedures and open-heart surgeries in the five years of the cardiology and cardiothoracic department’s establishment.

## Background

Cardiovascular disease (CVD) is a leading cause of morbidity and mortality worldwide, with significant variations in burden across different regions (1). In high-income countries such as the USA, CVD accounts for nearly one in every three deaths, largely driven by lifestyle factors such as obesity, smoking, and sedentary behavior. However, access to advanced cardiovascular care, preventive measures, and early interventions has led to declining mortality rates over the years (2). In contrast, sub-Saharan Africa (SSA) faces a rising burden of CVD due to a combination of infectious and non-communicable disease risk factors. While rheumatic heart disease (RHD) remains a major contributor to acquired heart disease due to untreated streptococcal infections, hypertension and ischemic heart disease are emerging as leading causes of heart failure and stroke (3,4,5). Unlike in developed nations, limited healthcare infrastructure, late diagnosis, and inadequate treatment options have resulted in high mortality rates and a disproportionate share of the global cardiovascular disease burden, accounting for approximately 80% of all CVD-related deaths worldwide across low- and middle-income countries (LMIC) (6,7). In Tanzania, the leading cardiovascular conditions include hypertensive heart disease (41%), valvular heart disease (18%), coronary heart disease (18%), peripartum cardiomyopathy (7%), and non-hypertensive dilated cardiomyopathies (6%) among adults, while congenital heart disease accounts for 34% of pediatric cases (8,9). In a study conducted at the Benjamin Mkapa Hospital (BMH) by Meda et al., the prevalence of coronary artery diseases (CAD) of any degree was at 24.4%—predominantly among men—while that of obstructive CAD was 18.3%, indicating a high burden in the central zone of Tanzania (10).

The availability of cardiac catheterization laboratories in SSA remains critically inadequate, with most countries operating far below the recommended benchmark of one laboratory per one million people (11). In Tanzania, only two publicly owned cardiac catheterization laboratories exist, each serving over 30 million citizens. Recognizing these challenges, the launch of a cardiovascular center at the BMH in central Tanzania was a crucial milestone in improving access to specialized cardiac care. Before its establishment in 2018, cardiovascular care was centralized primarily in Dar es Salaam, where the Jakaya Kikwete Cardiac Institute (JKCI) served as the main referral center for advanced cardiac care, including surgeries and interventional procedures. However, these services were often inaccessible to the majority of Tanzanians due to high costs, long waiting times, and geographical barriers (12). Outside Dar es Salaam, most regional and district hospitals lacked cardiologists, diagnostic tools such as echocardiography and cardiac catheterization labs, and essential medications for heart disease management. Primary healthcare facilities focused largely on infectious diseases, with noncommunicable diseases (NCDs) receiving less priority in funding and policy-making. As a result, many cardiovascular conditions were diagnosed late, often when complications such as stroke, heart failure, or sudden cardiac death had already occurred (13). The growing burden of CVDs, combined with limited healthcare infrastructure, highlighted the urgent need for improved cardiac care services, particularly in underserved regions like Dodoma.

The primary objective of establishing the cardiovascular center at the BMH was to bridge this gap in specialized cardiac services by providing accessible, high-quality care to patients in central Tanzania and beyond. The center aimed to offer advanced diagnostic and interventional cardiology services, cardiac surgeries, and comprehensive management of CVDs. Additionally, it seeks to promote research, capacity-building, and training of healthcare professionals, which are essential for sustainable health system strengthening. Here, we aim to evaluate the implementation and early outcomes of the cardiology and cardiothoracic program at the BMH (2019–2024) using a service evaluation approach, describing service development, procedure volumes, selected clinical outcomes, and lessons learned, to inform similar scale-up efforts in low-resource settings.

## Methods

### Study design

A retrospective descriptive analysis of the cardiology and cardiothoracic clinical practices performance and patient database at the BMH was conducted, covering the period from February 15, 2019, to August 30, 2024. Document review was used to describe the evolution of cardiology and cardiovascular services at the BMH.

### Study site

The BMH is a specialized tertiary healthcare facility located in Dodoma, Tanzania, offering a wide range of specialized services, including advanced cardiovascular care. As a teaching hospital affiliated with the University of Dodoma, the BMH plays a crucial role in medical training and research while serving as a key referral hub for central Tanzania. The hospital’s department of cardiology and cardiothoracic services handles a substantial patient load, with approximately 26,000 outpatient consultations and 2,000 inpatient admissions annually. In addition, the department performs around 7,200 thoracic echocardiograms and 8,000 resting electrocardiograms each year. Beyond routine diagnostics, more than 1,500 advanced procedures are conducted annually, including open-heart surgery, diagnostic coronary angiography (CAG), percutaneous coronary interventions (PCI), pacemaker implantations, exercise and Holter electrocardiograms, and ambulatory blood pressure monitoring (10).

### Study population and ethical clearance

The study population included both children and adults who underwent diagnostic and/or interventional procedures in the department of cardiology and cardiothoracic services during the study period. This study complied with the Declaration of Helsinki. A waiver of individual informed consent was granted by the Institutional Ethics Committee of the BMH, and all patient data were fully anonymized to ensure confidentiality.

### Data analysis

The characteristics of the study population were summarized using frequencies and proportions. Angiogram to PCI rate (%) was calculated as the total number of CAGs performed divided by the number of PCI procedures and multiplied by 100%. The analysis was guided by Donabedian’s structure, process outcome model, and the RE-AIM (Reach, Effectiveness, Adoption, Implementation, Maintenance) framework. Structure measures included infrastructure (cath-lab, CCU beds, medical equipment) and staffing (nurses, physicians, and cardiologists); process measures included procedural volumes; outcome measures included in-hospital mortality. For statistical analysis, the data were first captured into a Microsoft Excel spreadsheet and then imported into IBM SPSS Statistics for Windows, Version 26.

## Results

### The targeted population, disease burden, and expected outcomes

The Central Zone of Tanzania, served by the BMH, encompasses six regions: Dodoma, Singida, Tabora, Manyara, Morogoro, and Iringa, covering an estimated population of 12–15 million people. This largely rural and peri-urban population has historically faced severe gaps in access to advanced cardiovascular care, with the nearest cardiac catheterization laboratories and cardiothoracic surgical services located hundreds of kilometers away in Dar es Salaam or outside the country. Cardiovascular disease (CVD) burden in the region is rising, driven by increasing rates of hypertension (43.4%), diabetes (4.5%), obesity (29.9%), and RHD (6–21%), compounded by limited diagnostic capacity and delayed referrals (14,15). The rollout of cardiology and cardiothoracic services at the BMH, comprising cardiac catheterization, PCI, pacemaker implantation, congenital heart defect closure, and open-heart surgery, directly addresses this underserved need. By decentralizing advanced CVD care, reducing referral delays, and integrating workforce training with infrastructure expansion, the program is positioned to improve early diagnosis, timely interventions, and overall survival, while reducing out-of-pocket costs for patients who previously required overseas referrals.

### Transformative changes made at Benjamin Mkapa Hospital

The department of cardiology and cardiothoracic services has undergone significant transformations to enhance cardiovascular care, making it a key referral center in central Tanzania. One of the most notable advancements has been in infrastructure, with the development of a state-of-the-art catheterization laboratory to support interventional cardiology procedures such as right heart catheterization (RHC), diagnostic CAG, and PCI. The hospital has also established a dedicated 10-bed cardiac care unit (CCU) to provide intensive monitoring and management of critically ill cardiovascular patients. Additionally, two modern operating theaters have been set aside exclusively for cardiothoracic procedures, enabling the hospital to perform advanced heart and lung surgeries that were previously unavailable in the region.

In addition to infrastructure development, the BMH has significantly invested in modern cardiovascular equipment to enhance diagnostic accuracy and therapeutic interventions. The facility now boasts two heart-lung machines for open-heart surgeries, a fluoroscopic X-ray machine (C-arm), and a stress ECG system. Furthermore, the hospital has acquired four echocardiography (ECHO) machines and three electrocardiogram (ECG) machines for routine cardiac evaluations. To facilitate continuous heart rhythm monitoring, the hospital has also procured 10 Holter ECG monitors and 15 ambulatory blood pressure machines, improving the early detection and management of hypertension and arrhythmias.

A critical aspect of the hospital’s transformation has been the development of human resources for health, focusing on the training and expansion of its cardiovascular workforce. Over the years, the BMH has successfully trained and recruited six cardiologists, including two interventional cardiologists specializing in minimally invasive heart procedures. Additionally, the hospital now has two cardiothoracic surgeons and one cardiovascular surgeon capable of performing complex heart and lung surgeries. The surgical team is further supported by a skilled perfusionist and 10 specialized cardiac care unit nurses, ensuring high-quality perioperative, intraoperative, and postoperative care for cardiac patients. These advancements have been made possible through strategic collaborations with both local and international institutions. These include structured skill-transfer programs, super specialization fellowship training locally and abroad, in-service mentorship by visiting teams, and national-level accreditation processes by the Medical Council of Tanganika under the umbrella of the Ministry of Health.

Between 2019 and 2024, capacity building at the BMH followed a phased approach. In the early phase (2019–2021), structured skill-transfer programs were conducted during annual and biannual visits by interventional cardiology and cardiothoracic surgery teams from Kuwait, the Netherlands, and the USA, which facilitated specialized training, knowledge exchange, and the adoption of best practices in cardiovascular care. These visits provided hands-on procedural training, case-based discussions, and simulation exercises, enabling local staff to progress from observation to supervised lead operator roles. Parallel to this, selected cardiologists and surgeons were enrolled in 12–24-month subspecialty fellowships abroad (India, South Africa, and Russia). Returning from training, 2022 onward, in-service mentorship was reinforced through short, intensive visits by international teams every six months, focusing on skill gaps identified via case reviews and supplemented by remote telemedicine consultations. These coordinated efforts led to a marked increase in procedural volume, reduced reliance on visiting teams, and the establishment of sustainable cardiovascular services. A formal memorandum of understanding and partnership between the BMH and JKCI was set for in-house training programs, allowing staff to gain in-house hands-on experience from experts within Tanzania. Quarterly short-term intensive visits (1–2 weeks) every 3–4 months were implemented. Annual cardiovascular screening and treatment camps were conducted, and patients were successfully managed. Furthermore, complex procedures and implantation of devices among selected patients have been successful under the supervision of cardiologists from JKCI. In addition to clinical skills, the BMH and JKCI have extended their collaboration in research and the establishment of multicenter studies. These partnerships have played a crucial role in strengthening the hospital’s capacity to provide high-quality cardiac services and ensure sustainable growth in the field of cardiovascular medicine. Through these transformative changes, the BMH has positioned itself as a leading center for cardiovascular care in central Tanzania, significantly improving access to life-saving interventions and reducing the burden of heart diseases in the region.

### Financing of cardiovascular care

The financing of cardiac procedures relied on multiple funding sources, ensuring access to life-saving interventions for patients from diverse financial backgrounds. Approximately 10% of both adult and pediatric cardiac procedures were funded out-of-pocket by patients and their families. Another 10% of procedures, primarily pediatric cardiac surgeries, were fully financed by the government through the Ministry of Health, enabling children with congenital and acquired heart diseases to receive specialized treatment without financial burden.

The majority of procedures, accounting for nearly 70%, were fully covered by the National Health Insurance Fund (NHIF). These were predominantly diagnostic CAG, PCI, and pacemaker implantations, which were essential for managing ischemic heart disease and cardiac arrhythmias. The remaining procedures, mostly open-heart surgeries, were co-financed by NHIF and patients. In these cases, the insurance fund covered 70% of the total cost, while patients contributed the remaining 30%, making complex surgical interventions more financially accessible. This structured financing model played a crucial role in ensuring equitable access to cardiac care while supporting the hospital’s sustainability in delivering specialized cardiovascular services.

### Number of procedures performed between 2019 and 2024

On analyzing our catheter laboratory data, a total of 1,313 cardiovascular procedures were performed at the BMH between 2019 and 2024. As shown in Table 1, these procedures were categorized into three main groups: cardiac catheterization procedures for adults (1,215), cardiac catheterization procedures for children (55), and cardiac surgeries (43). Among adult cardiac catheterization procedures, diagnostic CAG was the most common, accounting for 88.8% of cases, followed by PCI (9.5%), where the angiogram-to-PCI rate was 10.6%. In pediatric cardiac catheterization procedures, RHC was performed in 29% of cases, while patent ductus arteriosus (PDA) closure was the most frequently conducted cardiac surgery, representing 42% of all surgical interventions. The frequency of procedures in the first to third year was lower compared to the fourth and fifth year of operation (Supplementary Figure 1) in both PCI and open-heart surgeries.

**Table 1:** Total number of procedures performed from 2019 to 2024.


PROCEDURE	FREQUENCY N (%)

**Adult cardiac catheterization procedures**	

Coronary angiography	1081 (88.9)

Percutaneous coronary intervention	115 (9.5)

Pacemaker implantation	19 (1.6)

**Total**	**1215 (100)**

**Pediatric cardiac catheterization procedures**	

Diagnostic right heart catheterization	16 (29.1)

ASD device closure	10 (18.1)

PDA device closure	18 (32.7)

Pulmonary valve valvuloplasty	11 (20)

**Total**	**55 (100)**

**Cardiac surgeries**	

PDA closure	18 (41.9)

ASD closure	8 (18.6)

TOF closure	3 (7)

VSD closure	4 (9.3)

Pulmonary artery banding	3 (7)

Mitral valve replacement	1 (2.3)

Aortic valve replacement	1 (2.3)

Coronary artery bypass graft	5 (11.6)

**Total**	**43 (100)**


ASD: Atrial septal defect; PDA: Patent ductus arteriosus; TOF: Tetralogy of Fallot; VSD: Ventricular septal defect.

### Cardiac catheterization procedures for pediatric and adult populations

**Figure 1:**
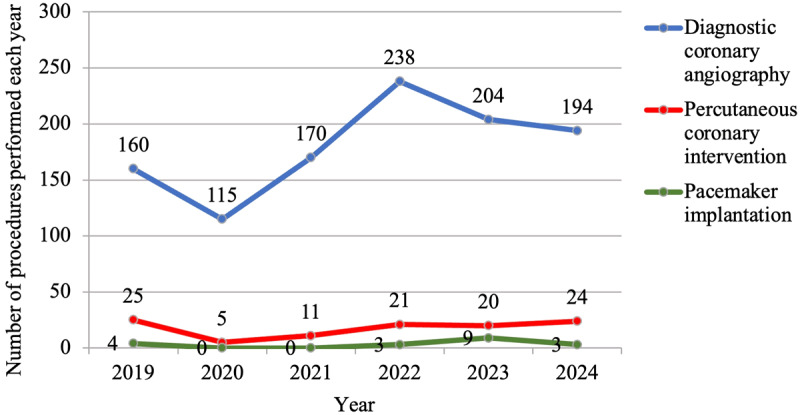
Number of adult cardiac catheterization procedures performed from 2019 to 2024.

Since the installation of the catheterization laboratory in 2019, diagnostic CAG procedures were initially performed by a single in-house interventional cardiologist with support from the JKCI. From 2020 onward, the enrollment of additional cardiologists contributed to a steady increase in the number of procedures performed annually (Figure 1). This growth reflects improved local capacity and reduced dependence on external expertise. Following the installation of the catheterization laboratory in 2019, patients presenting with acute myocardial infarction who were initially referred to the JKCI due to limited local capacity for emergency PCI were attended to at the BMH. During this period, our team, with support from JKCI, performed PCI exclusively on patients with chronic ischemic heart disease. However, by 2020, after a year of capacity building and specialized training, our team became fully equipped to independently perform revascularization procedures for both acute myocardial infarction and chronic ischemic heart disease, significantly improving access to timely interventions and patient outcomes. Following three years of capacity building, in 2022, our team successfully developed the expertise to perform both single and dual-chamber pacemaker implantations.

**Figure 2:**
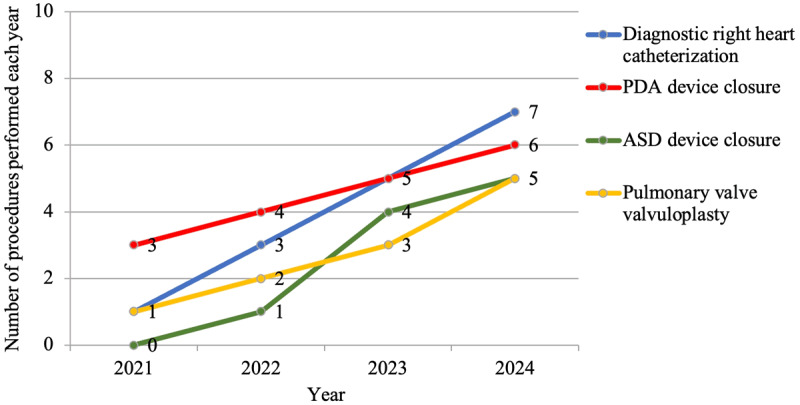
Number of pediatric cardiac catheterization procedures performed from 2021 to 2024. ASD: Atrial septal defect; PDA: Patent ductus arteriosus.

The initiation of pediatric cardiac catheterization procedures faced a two-year delay due to the lack of local expertise (Figure 2). From 2021 onward, these procedures were conducted once or twice annually with the support of visiting specialists from the Netherlands, Kuwait, and the USA. Currently, two pediatric interventional cardiologists are undergoing specialized training, and upon completion of their program, the hospital anticipates a significant increase in the number of pediatric catheterization procedures performed annually, ensuring better access to life-saving interventions for children with congenital and acquired heart diseases.

### Cardiac surgeries

Since 2021, the BMH has been offering cardiac surgeries for both pediatric and adult patients, initially performed exclusively by visiting experts from Kuwait and the Netherlands. These international collaborations have played a crucial role in building local expertise and enhancing the hospital’s capacity in advanced cardiovascular procedures. As a result of this knowledge transfer and skill development, the hospital has been independently performing cardiac surgeries since 2023. This transition is reflected in the steady annual increase in the number of procedures, demonstrating the hospital’s growing surgical capability and commitment to expanding cardiovascular services as shown in Table 2.

**Table 2:** Number of Cardiac Surgeries that were Performed from 2019 to 2024.


PROCEDURE	YEAR				TOTAL

	2021	2022	2023	2024	

PDA closure	1	5	9	3	18

ASD closure	3	1	3	1	8

TOF closure	0	0	3	0	3

VSD closure	0	0	4	0	4

PA banding	0	0	3	0	3

CABG	0	0	3	2	5

Mitral valve replacement	0	0	0	1	1

Aortic valve replacement	0	0	0	1	1

Total	4	6	25	8	43


ASD: Atrial septal defect; CABG: Coronary artery bypass graft; PDA: Patent ductus arteriosus; TOF: Tetralogy of Fallot; VSD: Ventricular septal defect; PA: Pulmonary artery.

### In-hospital outcomes

In our study, we mainly assessed the in-hospital mortality. Among 115 patients who underwent PCI, four (3.5%) deaths were recorded, the majority of whom had suffered from acute myocardial infarction. Despite receiving timely and successful revascularization, these patients succumbed to complications of the disease, such as severe heart failure and arrhythmias. Additionally, among the 43 patients who underwent cardiac surgery, three (6.9%) deaths were attributed to immediate surgical complications following open-heart procedures. The most likely cause was postoperative hemorrhage, a known risk factor in complex cardiac surgeries. The rates of in-hospital mortality highlight both the hospital’s progress in delivering advanced cardiovascular care and the need for continuous improvements in surgical techniques, postoperative monitoring, and intensive care management to further enhance patient survival rates.

## Discussion

The development of cardiovascular services at the BMH has led to significant advancements in the management of heart diseases in central Tanzania, with over 1,300 procedures performed between 2019 and 2024, improving healthcare equity and strengthening the healthcare system. This aligns with the global efforts to decentralize specialized care and strengthen regional health systems as emphasized by the World Health Organization (WHO) (16). In the initial three years, the hospital primarily relied on annual skill transfer programs facilitated by local cardiologists from JKCI and visiting experts from Kuwait, the Netherlands, and the USA. This period was characterized by a relatively low number of procedures, as local expertise was still being developed and complex interventions were primarily performed during training programs and treatment camps. As observed in similar capacity-building initiatives across SSA (17), these early interventions served as critical stepping stones. The marked increase in procedural volume in the last two years signifies a successful transition from dependency to autonomy. This progress correlates with the recruitment of trained specialists and the return of Tanzanian health professionals trained in interventional cardiology and cardiac surgery abroad. Procedures such as CAG, pacemaker insertions, and open-heart surgeries are now routinely performed, which is a remarkable achievement given the initial absence of such services in the region. The SSA region faces a severe shortage of cardiac catheterization laboratories, with most countries falling far below the recommended minimum of one catheterization laboratory per 1 million people (11,18). In Tanzania, only two public catheterization laboratories exist, each serving over 30 million citizens, highlighting a critical gap in access to lifesaving cardiovascular care. This signifies the effectiveness of targeted workforce development and infrastructure development as a core strategy for sustainable health system strengthening (19).

The institution observed that among adults, the most frequently conducted interventions were CAG and PCI, followed by permanent pacemaker implantation. In the pediatric population, a high number of diagnostic RHCs were observed, along with PDA device closures and surgical repairs for both PDA and atrial septal defects. The pattern of conditions managed and procedures performed is consistent with trends reported by other regional cardiovascular centers, reflecting similar epidemiological profiles and service needs (6,11). The angiogram-to-PCI rate during the study period was 10.6%, which appears lower than international benchmarks of 35–60% in mature programs. However, findings from early-stage interventional programs in resource-limited settings and the East African region suggest similar rates of 12–15%, where a significant proportion of referrals present late, have non-obstructive CAD, or were initially evaluated for congenital or valvular pathology (20). As procedural volume increases and indications become more refined, this rate is expected to rise. Furthermore, we emphasize that CAG was initially performed primarily for diagnostic purposes, as PCI services were rolled out more cautiously. The rate of in-hospital mortality for PCI (3.5%) and cardiac surgery (6.9%) was mostly observed among emergent rather than elective procedures. Most of the deaths resulted from the severity of underlying cardiac conditions, particularly in patients with acute myocardial infarction presenting with cardiogenic shock, as well as surgical complications, including postoperative hemorrhage following open-heart procedures. These findings suggest strengthening of the emergency response team, underscoring the need for continued advancements in surgical techniques, perioperative care, patient monitoring, and improvement of critical care post-surgery to further optimize clinical outcomes.

The introduction of cardiovascular services at the BMH has been met with several setbacks that hinder optimal care delivery. One major issue is the low number of patients presenting with acute myocardial infarction (MI) for primary PCI. This is largely due to referring physicians’ lack of a high index of suspicion for coronary artery disease, insufficient knowledge about the disease burden and its clinical presentation, and limited awareness of the management options available in the region (13). Consequently, the majority of procedures performed at the hospital have been elective, particularly for unstable angina, rather than emergency interventions for acute myocardial infarction. A key diagnostic tool for early detection of myocardial ischemia, the ECG, is frequently unavailable in regional and primary healthcare facilities, making it difficult to diagnose acute myocardial infarction in adult patients with chest pain. Even in centers that do have ECG machines, physicians often lack basic training in ECG interpretation, especially for life-threatening emergencies such as coronary artery disease. This results in delayed diagnoses and missed opportunities for early revascularization, which is critical for improving patient outcomes within the recommended door-to-balloon time (13,21). As a result, the existing catheterization laboratory in the region is underutilized, as timely interventions are not always possible. Although recent efforts by the Tanzania Ministry of Health have focused on supporting the management of NCDs, including cardiovascular diseases, challenges remain as in other parts of SSA (22). These efforts include budgetary allocations, procurement of equipment, and the training of healthcare professionals. The JKCI, as a sister institution, has played a pivotal role in providing in-house training and disseminating new knowledge for cardiovascular disease management across the country. However, the sustainability of catheterization laboratory services continues to be a significant challenge. This is primarily due to a shortage of qualified healthcare professionals, including cardiologists, cardiac surgeons, cardiovascular technicians, perfusionists, and cardiac anesthetists. In addition, the lack of a comprehensive health financing system to support patients who cannot afford the costs of treatment is a major barrier. High costs for catheterization laboratory consumables and supplies, combined with bureaucratic hurdles in government procurement systems, further complicate the situation. The infancy of backup services, such as open-heart surgery, poses a great risk, especially in cases of intra-procedural complications during catheterization. The American Heart Association (AHA) recommendations advocate a minimum procedural volume for proficiency (≥ 75 PCI cases per operator/year; ≥ 200 PCI cases per institution) (23). However, many centers in LMICs operate below these thresholds. To overcome the challenges of being a low-volume center (< 50 PCI per year), the BMH has implemented a structured competency and credentialing pathway adopted from the WHO’s Package of Essential Non-communicable diseases intervention (PEN-Plus strategy) in LMICs. Capacity is built through intensive supervision by visiting local and international teams, structured mentorship, and the reintegration of specialists trained abroad, all accredited by the Ministry of Health. Furthermore, the BMH has made significant strides in addressing the gaps in cardiovascular care by training more cardiologists, cardiac surgeons, cardiovascular technicians, perfusionists, and cardiac anesthesiologists to strengthen local expertise. Moreover, the hospital is now equipped with advanced equipment, including a heart-lung machine and an extracorporeal membrane oxygenation (ECMO) machine, signaling its commitment to providing high-quality cardiovascular services. Based on our experience we recommend that overcoming the challenges in LMICs will require (i) adopting staged implementation with clear structure-process-outcome indicators, (ii) implementing formal credentialing and mentorship pathways combining visiting expert supervision, structured fellowships, (iii) establishing a routine registry for prospective monitoring of procedural outcomes and complications, and (iv) continued investment in human resources, infrastructure, and a more robust health financing system to ensure the sustainability and accessibility of these services (3). Our findings are consistent with other program evaluations from the region. The Uganda Heart Institute’s catheterization lab experience emphasized phased skill transfer and quality monitoring during scale-up (11). Furthermore, similar program evaluations in Kenya and Uganda report low initial angiogram PCI rates, progressive local capacity building, and the importance of partnerships for sustainable growth (6,17). Strategic partnerships with the Cardiac Surgery Intersociety Alliance, the World Heart Federation, and non-governmental organizations such as Madaktari Africa to further drive surgical access, workforce development, and national CVD advocacy, ensure that service growth remains sustainable and globally benchmarked (6). This study has several limitations. We performed a retrospective descriptive analysis for service evaluation relying on hospital records, which had missing data on demographics, cardiovascular risk factors, comorbidities, disease severity, and laboratory data, limiting our statistical analysis. Procedural outcomes were mainly reported as mortality, with other complications (vascular access complications, peri-procedural stroke, acute kidney injury, arrhythmias, postoperative infections) not systematically documented. The relatively small procedural volumes reflect the early phase of service implementation, further affected by COVID-19, which delayed referrals, procedures, and international training activities. The absence of long-term follow-up and comprehensive benchmarking metrics (e.g., door-to-balloon time, procedural success rates, patient satisfaction) restricts comparison with global standards. Nonetheless, the BMH has addressed these gaps by establishing prospective patient registries to enhance future research output.

## Conclusion

The establishment of cardiovascular care at the BMH has resulted in notable progress in the diagnosis and treatment of heart diseases, with a significant increase in the number of procedures performed in recent years. The growth of local expertise, fueled by specialized training and the recruitment of skilled professionals, has contributed to the hospital’s ability to independently handle advanced cardiovascular interventions. However, challenges such as limited awareness among referring physicians, inadequate diagnostic tools in primary healthcare facilities, and a shortage of trained personnel remain significant barriers to optimal care delivery. Furthermore, the lack of a comprehensive health financing system and challenges in procurement and supply chain management continue to impede the sustainability of cardiovascular services. Despite these obstacles, the BMH has planned to continue addressing these gaps and is committed to further strengthening its capacity to deliver high-quality care through continued investment in human resources, infrastructure, and healthcare financing. With ongoing support from national and international collaborations, the hospital is poised to enhance cardiovascular care for the population in central Tanzania and beyond.

## Additional File

The additional file for this article can be found as follows:

10.5334/gh.1488.s1 Supplementary Figure 1. Frequency of procedures performed in years 1–3 and 4–5.
