# Supplementary figures and images for: Transformation of Cardiology and Cardiothoracic Services at Benjamin Mkapa Hospital, Tanzania: Findings and Experiences from 1,313 Cardiovascular Procedures in Five Years
Source: Glob Heart. 2025 Oct 17;20(1):98. doi: 10.5334/gh.1488 (PMC12533416; doi:10.5334/gh.1488)

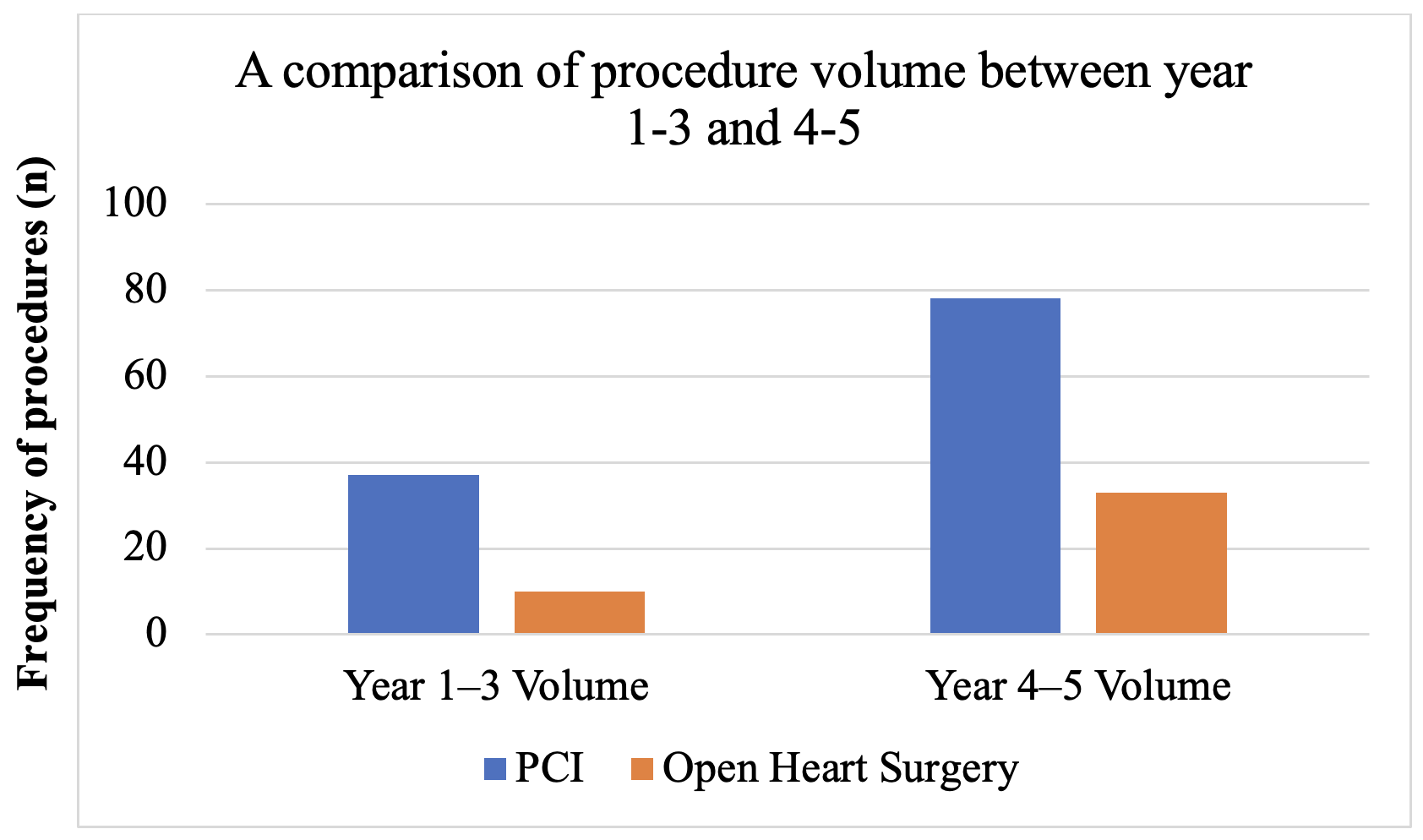

Supplement: Supplementary Figure 1. — Frequency of procedures performed in years 1–3 and 4–5. [file gh-20-1-1488-s1.png]
